# Targeting PI3K*γ*/AKT Pathway Remodels LC3‐Associated Phagocytosis Induced Immunosuppression After Radiofrequency Ablation

**DOI:** 10.1002/advs.202102182

**Published:** 2022-01-17

**Authors:** Xiaodi Liu, Wenyue Zhang, Yanni Xu, Xiaolin Xu, Qiongchao Jiang, Jingliang Ruan, Ye Wu, Yingshi Zhou, Phei Er Saw, Baoming Luo

**Affiliations:** ^1^ Department of Ultrasound Sun Yat‐sen Memorial Hospital Sun Yat‐sen University Guangzhou 510120 China; ^2^ Guangdong Provincial Key Laboratory of Malignant Tumor Epigenetics and Gene Regulation Sun Yat‐sen Memorial Hospital Sun Yat‐sen University Guangzhou 510120 China

**Keywords:** LC3‐associated phagocytosis, macrophage, radiofrequency ablation

## Abstract

Residual tumors after insufficient radiofrequency ablation (IRFA) shows accelerated progression and anti‐PD‐1 resistance. It is also reported that macrophages infiltrating into residual tumors leads to anti‐PD‐1 resistance. Elements of autophagy have been detected to conjugate LC3 to be increasingly expressed in residual tumors. The underlying mechanisms between LC3 and macrophages are aimed to be investigated, and explore further ways to enhance immunotherapy in treating residual tumors. In mice models and patients, macrophages demonstrate increased infiltration into residual tumors, especially surrounding the ablated zone. Single‐cell transcriptome demonstrates enhancement of immunosuppression function in macrophages after IRFA. It is shown that macrophages engulf heat‐treated cells through LC3‐associated phagocytosis (LAP), enhance IL‐4 mediated macrophage programming through the PI3K*γ*/AKT pathway, and suppress T cell proliferation. Blockade of the PI3K*γ*/AKT pathway enhances the antitumor activity of PD‐1 blockades, inhibits malignant growth, and enhances survival in post‐IRFA models. In conclusion, in mice models and patients, macrophages demonstrate increased infiltration around ablated zones in residual tumors. Blockade of the PI3K*γ*/AKT pathway suppresses the growth of residual tumors in subcutaneous and orthotopic models. The results illustrate the translational potential of PI3K*γ* inhibitors to enhance anti‐PD‐1 therapy for the treatment of residual tumors after IRFA.

## Graphical abstract

In hepatocellular carcinoma treated by insufficient radiofrequency ablation, LC3‐associated phagocytosis (LAP) is induced in macrophages, where the macrophages engulf dying tumor cells. Cholesterol efflux during LAP enhances IL‐4 mediated programming and activates the PI3K*γ*/AKT pathway. Cytokines including CCL7 and IL‐10 are produced after LAP. Simultaneously, inhibition of LAP and blockade of the PI3K*γ*/AKT pathway remodels the immunosuppressive state of macrophages.

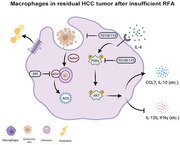

## 1 Introduction

Radiofrequency ablation (RFA), a thermal ablation technique, is one of the main curative therapies for early‐stage hepatocellular carcinoma (HCC).^[^ ^1^, ^2^ ^]^ RFA induces the heating of tumor tissue as well as surrounding liver tissue, leading to tumor necrosis. RFA treatment is effective and minimally invasive, with a low prevalence of complications.^[^ ^3^, ^4^ ^]^ In some cases, however, patients who have undergone RFA treatment experience a phenomenon called “insufficient RFA (IRFA)”, where the tumors did not achieve sufficiently high temperatures required for treatment due to their size or location.^[^ ^5^ ^]^ The residual viable tumor cells could be a main source of recurrence and metastasis. Recently, rapid and aggressive recurrence and metastasis in patients with HCC after IRFA have been reported by various studies, including ours.^[^ ^6^, ^7^, ^8^, ^9^ ^]^ However, the detailed mechanisms of the onset of tumor aggression after IRFA have yet to be fully elucidated.

The non‐malignant cells of the tumor microenvironment (TME) can comprise >50% of the mass of primary tumors and their metastases. They were also shown to perform complicated roles in the progression of residual tumors. Several studies on preclinical animal models have demonstrated that localized tumor ablation by RFA could release tumor antigens and induce systemic T‐cell–mediated antitumor immunity.^[^ ^10^, ^11^, ^12^, ^13^, ^14^ ^]^ However, the RFA‐induced immune responses were insufficient to prevent tumor recurrence. A study has presented the infiltration of macrophages into the border zone between residual tumors and ablated zones,^[^ ^15^ ^]^ and another study has demonstrated that IRFA was able to induce anti‐PD‐1 resistance due to the existence of macrophages.^[^ ^14^ ^]^ However, the mechanisms of the immunosuppressive function of macrophages have not been elucidated.

RFA leads to plenty of dying cells in situ. However, there are few studies investigating the role of dying cells in the aggressive state of residual tumors after IRFA. Previous studies have shown the non‐canonical function of autophagy proteins of engulfing dying cells through lipidation of LC3/GABARAP‐family proteins in macrophages, dendritic cells (DCs), and epithelial cells.^[^ ^16^, ^17^, ^18^ ^]^ LC3‐associated phagocytosis (LAP) serves as an innate defense mechanism against invading microorganisms including bacteria, fungi, and parasites.^[^ ^19^, ^20^ ^]^ Studies have shown that the LAP pathway also induces inflammatory tumor immune tolerance by regulating polarization of TAM into anti‐inflammatory M2 cells.^[^ ^21^, ^22^ ^]^ We previously found that LC3 proteins were upregulated after IRFA.^[^ ^7^ ^]^ Further studies are required to unravel whether macrophages undergo LAP in residual tumors, and whether remodeling of the TME induced by LAP could promote the therapeutic effect of anti‐PD‐1 therapy.

In this study, we demonstrated that macrophages were recruited after IRFA and underwent LAP. LAP in macrophages promoted IL‐4‐mediated macrophage programming, activated the PI3K*γ*/AKT pathway, and expressed anti‐inflammatory cytokines that induced immune suppression. We found that targeting PI3K*γ*, a selective inhibitor for lymphocytes, could reprogram the infiltrating macrophages in residual tumors and promote anti‐PD‐1‐mediated tumor regression. Based on our results, we would recommend new combination strategies with PI3K*γ* inhibitors and anti‐PD‐1 to treat residual tumors after IRFA.

## 2 Results

### 2.1 The Number of Macrophages Increases in Residual Tumors after IRFA

**Figure 1:**
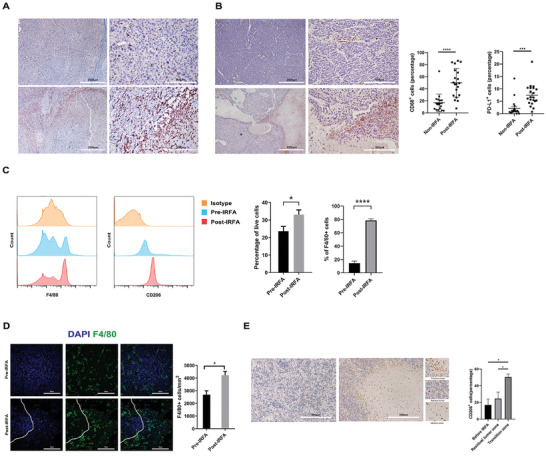
The number of macrophages increases in the residual tumors after IRFA. A,B) Representative immunohistochemistry of CD68 and PDL1 in HCC sections in both normal tumors and tumors after IRFA in clinical patients was shown (n = 21 for the RFA group, n = 19 for the non‐RFA group, **** *p* < 0.0001 for the CD68 expression; *** *p* < 0.001 for the PDL1 expression by unpaired *t*‐test). Scale Bar  =  2000 µm and 500 µm. C) The proportion of F4/80^+^ and CD206^+^ cells in tumor tissue of mice after treatment was quantified by flow cytometry (n = 4, *p = 0.0496 for F4/80 expression by unpaired *t*‐test; ****p < 0.0001 for CD206 expression by unpaired *t*‐test;). D) Representative IF of F4/80 in tumor sections of mice. The zone with rare DAPI represented the ablated zone, with macrophage infiltration into the ablated zone. Scale Bar  =  100 µm (n = 5, *p = 0.0067 by unpaired *t*‐test). E) Representative immunohistochemistry of CD206 in tumor sections of mice. The sections were divided into 3 parts, residual tumors, ablated zone, and borderline zone, where the CD206^+^ cells accumulated in the borderline (n = 4, *p = 0.0381 by paired *t*‐test). Scale Bar  =  500 µm.

To study whether residual tumors recruited macrophages after IRFA, we retrospectively studied a cohort of patients with liver cancer in our hospital. Twenty‐one patients who received tumor resection after RFA due to recurrence were assigned into the RFA group, whereas 19 patients who received primary tumor resection without RFA were assigned into the non‐RFA group. The characteristics of patients were summarized in Tab S1, Supporting Information, where there were no differences according to the clinical, biological, and histological features. In the specimens, a higher number of tumor‐infiltrating CD68^+^ macrophages was observed in the RFA group compared with that of the non‐RFA group, and the macrophages demonstrated increased infiltration around the ablated zone (**Figure** **1**A). Whereas in recent reports, recurrent tumors did not show higher infiltration of macrophages.^[^ ^23^, ^24^ ^]^

We also detected PD‐L1 expression and found that it was higher in the RFA group compared with that of the non‐RFA group, indicating that anti‐PD‐1 therapy might be effective in treating residual tumors after IRFA (Figure 1B). However, it has been reported that the infiltration of macrophages contributed to anti‐PD1 resistance in residual tumors, but the way to enhance the efficacy of anti‐PD1 therapy in treating residual tumors is still unknown.^[^ ^14^ ^]^

To systematically understand the aggressiveness and immunosuppressive states after IRFA, we established the IRFA animal model. Subcutaneous tumors were seeded in both flanks of mice, one tumor was excised and analyzed, the status of cells before IRFA was presented, and this was defined as the non‐treated control (NC) group. The other tumor underwent IRFA, and was excised after seven days, the status of cells after IRFA was presented, and this was defined as the IRFA group. H&E staining was performed to confirm the success of IRFA (Figure S1A, Supporting Information). Using IHC staining of tumor sections with Ki67, there was a higher proportion of cycling tumor cells after IRFA compared with cells before IRFA, and the cycling cells after IRFA were predominantly located in the transition zone (TZ) between ablated zones and the residual tumors (Figure S1B, Supporting Information), which was consistent with previous studies, showing the aggressive states of tumor cells.^[^ ^9^ ^]^

Through multi‐marker flow cytometry, we detected a higher proportion of macrophages, especially M2 macrophages, after IRFA in mice models (Figure 1C). Further immunofluorescence (IF) showed dominant expression of macrophages in the TZ (Figure 1D,E), indicating that there might be some crosstalk between dying cells and macrophages. In addition, a higher proportion of Tregs was detected in mice models, indicating an immunosuppressed state after IRFA (Figure S1C, Supporting Information).

### 2.2 Single‐Cell Transcriptome Identifies the Immunosuppression Function of Macrophages After IRFA

With the IRFA animal models, we collected tumor cells in the NC and IRFA groups. In both groups, freshly isolated tumors underwent rapid dissociation and were immediately subjected to fluorescence activated cell sorter (FACS) to obtain viable single cells. Then, we prepared cDNA from individual cells, constructed a single‐cell RNA‐seq library, and performed next‐generation sequencing (NGS). A total of 10 690 cells were analyzed in the NC group, while 9672 cells were analyzed in the IRFA group. 99.85% of cells in the NC group and 99.7% of cells in the IRFA group passed quality control. The average number of mapped reads per cell was about 8000 for the NC group and 7000 for the IRFA group. The median genes per cell were 726 and 1038 for the NC and IRFA groups, respectively.

**Figure 2:**
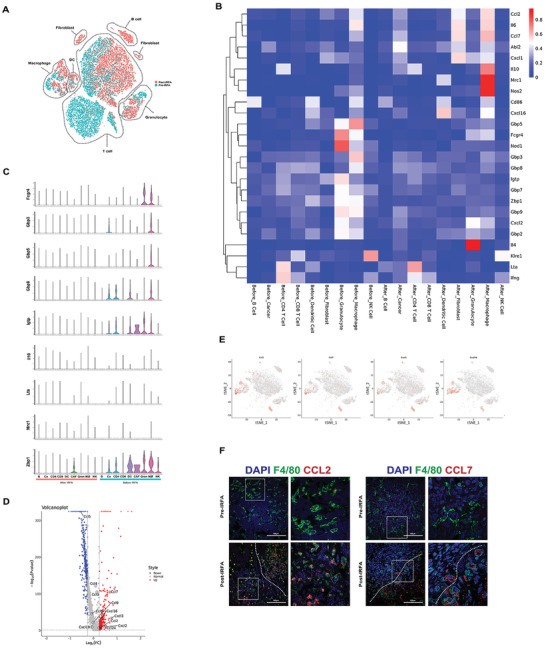
Macrophages after IRFA represent the immunosuppression phenotype. A) T‐SNE plot using scRNA‐seq data from cells sorted from normal tumors and tumors 7 days after IRFA. Cells from normal tumors served as the control. Control and IRFA samples were pooled. B) Expression in different clusters of immune response mRNA in tumors from tumor cells before and after IRFA. C) Violin plots showing the gene expression probability distribution of immune response. D) Volcano plot showing differential expression of chemokines between two lines (after / before IRFA). Genes with statistically significant differential expression (≥1.2‐fold, *p* < 0.05) located in the top right and left quadrants. E) Feature plot of CCL2, CCL7 expression across cell clusters after IRFA identified in Figure 2A. F) Fluorescence microscopy images illustrating CCL2, CCL7 (red) expression with DAPI nuclear (blue) and F4/80 (green) counterstain in residual tumors. Scale Bar  =  100 µm.

For non‐malignant cells, we annotated the clusters as T cells, macrophages, DCs, granulocytes, B cells, and fibroblasts, based on specific profiles of genes previously established to define these cells (Figure **2**A). It has been known that M2‐type macrophages are hallmarks of immune therapy in many kinds of tumors. We investigated the cytokine profiles of macrophages after IRFA. Mrc‐1, IL‐10 (the genes associated with M2 markers) increased after IRFA, while Gbp3, Gbp5, Fcgr4 (the genes related to innate immunity), Nod1 (the genes related to antigen presentation) decreased after IRFA (Figure 2B,C). In addition, the expression of CCL2, CCL7, CXCL1, CXCL2, CXCL16, and CCL24 was higher compared with normal tumors (Figure 2D). Among these chemokines, CCL2 was shown to be correlated to residual tumor progression.^[^ ^14^ ^]^ In our study, we found CCL2 and CCL7, known as immunosuppression‐related chemokines, to be dominantly expressed in macrophages, which was confirmed by IF (Figure 2E,F).^[^ ^25^, ^26^ ^]^ We searched the TCGA gene expression for chemokines indicative of progression‐free survival in HCC. The expression of the receptor of CCL2 and CCL7, CCR2, showed a correlation with disease‐free survival (DFS), and there was also a strong positive association between CCR2 expression and immunosuppressive markers such as CTLA4, PDCD1, TIMD‐4, and LAG3 (Figure S2, Supporting Information). These results indicated that after IRFA, the macrophages recruited monocytes into the residual tumors and monocytes switched to an immunosuppressive state, which might hinder the immune response after IRFA.

### 2.3 Macrophages Engulf Heat‐Treated Tumor Cells

Previously, we concluded that after IRFA, macrophages gathered around the TZ (Figure 1D). To investigate the relationship between heat‐treated cells and macrophages, we first established a model of heat‐treated tumor cells according to previous studies.^[^ ^9^, ^27^ ^]^ Hepa1‐6 cells were subjected to 60 °C for 10 min, and then stained with PI / Annexin V. The heat‐treated tumor cells showed significantly higher proportions of Annexin V and PI‐positive cells compared with tumor cells at 37 °C (Figure S3A, Supporting Information). The heat‐treated cells were further evaluated by a cell‐membrane impermeant nuclear fluorochrome, Ethidium Homodimer 1 (EthD‐1) and Cleaved‐Caspase‐7 (Figure S3B, C, Supporting Information).

**Figure 3:**
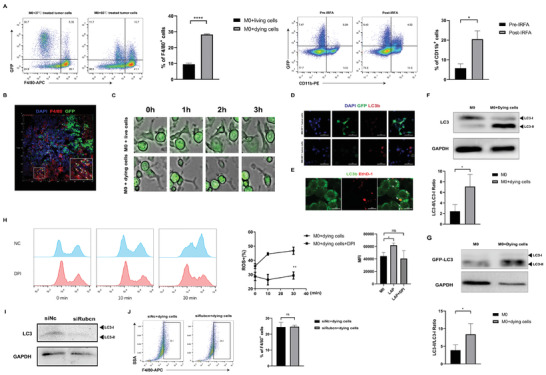
Macrophages engulf heat‐treated tumor cells through LAP. A) Representative flow cytometry plots illustrating macrophage phagocytosis of heat‐treated GFP‐transfected Hepa1‐6 cells in vitro and in vivo (n = 3, ****p = <0.0001 by unpaired *t*‐test). B) 3D fluorescence microscopy images illustrating the location of GFP^+^ tumor cells (green) with DAPI nuclear (blue) and F4/80 (red) counterstain in residual tumors (n = 3, *p = 0.0363 by unpaired *t*‐test). C) 60 °C‐treated and 37 °C‐treated Hepa1‐6 cells were stained by a membrane dye wheat germ agglutinin (WGA) (Arrows indicated), the process of tumor cell engulfment by macrophages was detected by high‐content microscopy. D) Representative confocal images from murine BMDMs, incubated with 37 and 60 °C‐treated GFP transfected Hepa1‐6 tumor cells and immunostained with LC3B antibody (red). Scale Bar  =  50 µm; E) Representative confocal images of Ethd‐1‐labeled Hepa1‐6 tumor cells (red) in murine BMDMs expressing GFP‐LC3 (green). Scale Bar  =  50 µm. F) Lysates from murine BMDMs were immunoblotted with antibodies to LC3 (n = 3, *p = 0.0464 by paired *t*‐test). G) Lysates from murine BMDMs expressing GFP‐LC3 were immunoblotted with antibodies to GFP (n = 3, *p = 0.0131 by paired *t*‐test). H) BMDMs engulfing dying cells were stained with CM‐H2DCFDA. Flow cytometry was performed to assess global ROS production. BMDMs were treated with or without DPI (10 µm) 1 h prior to stimulation with dying cells. Data shown are the percentages of ROS^+^ cells (left panel) and the mean fluorescence intensity (MFI) (right panel) of total cells (n = 3, **p = 0.0084 by unpaired *t*‐test, *p = 0.0108; ns = 0.6396 by unpaired *t*‐test). I) Lysates from murine BMDMs transfected with siRUBCN were immunoblotted with antibodies to LC3. J) Representative flow cytometry plots illustrating macrophage phagocytosis of heat‐treated dying cells (n = 3, ns = 0.9040 by unpaired *t*‐test).

Then, we extracted bone marrow‐derived macrophages (BMDMs) and induced them to mature. Flow cytometry was used to determine the purity of the macrophages (Figure S3D, Supporting Information). We applied a Hepa1‐6 cell line that stably expresses fluorescent GFP (GFP expressing Hepa1‐6 cells). The BMDMs were stimulated with heat‐treated GFP‐transfected tumor cells. Flow cytometry identified increased numbers of BMDMs engulfing GFP^+^ heat‐treated tumor cells compared with untreated cells (25.87% compared with 10.46%) (**Figure** **3**A). Furthermore, we labeled living cells with Calcein‐AM, dying cells with Ethd‐1, and detected that only a small number of macrophages engulfed living cells (Figure S3E, Supporting Information). Furthermore, we established the GFP^+^ Hepa1‐6 tumor‐bearing mouse model. The mice underwent IRFA and were euthanized after 24 h. Through flow cytometry, we found that macrophages demonstrated increased engulfment of GFP^+^ tumor cells in mice undergoing IRFA compared with untreated tumor‐bearing mice (20.49% compared with 5.77%) (Figure 3A). In tumor sections, GFP^+^ tumor cells overlapped the F4/80^+^ macrophages under 3D confocal microscopy (Figure 3B). The process of phagocytosis of heat‐treated cells by macrophages was also observed by high‐content microscopy. The macrophages (the irregular cells with none or little GFP fluorescence) barely engulfed normal tumor cells (the circular cells with bright GFP fluorescence, arrowhead) but engulfed heat‐treated cells (the circular cells with bright GFP fluorescence, arrowhead) in 3 h (Figure 3C, Videos S1 and S2, Supporting Information). These results indicated that macrophages engulfed dying tumor cells after IRFA.

### 2.4 Macrophages Engulf Dying Tumor Cells through LC3‐Associated Phagocytosis

It has been reported that macrophages engulf apoptotic, necrotic, and RIPK3‐dependent necrotic cells through LAP.^[^ ^28^ ^]^ We investigated whether IRFA induced LAP. Through a confocal microscope, we found that macrophages stimulated with heat‐treated tumor cells expressed higher levels of LC3 proteins, whereas normal tumor cells did not induce high expression of LC3 in macrophages and were hardly engulfed by macrophages (Figure 3D). With GFP‐LC3‐transfected macrophages, we detected that heat‐treated tumor cells resided in an LC3^+^ compartment 2 h after the incubation (Figure 3E, arrowheads). After treatment with heat‐treated cells, a significant increase in LC3‐II to LC3‐I was observed through WB (Figure 3F). We detected low levels of lipidated LC3 from both untreated and heat‐treated tumor cells, indicating that the LC3‐I to LC3‐II conversion detected in macrophages was induced by the engulfment of dying tumor cells (Figure S3F, Supporting Information). To further confirm our hypothesis, GFP‐LC3‐transfected macrophages were stimulated with dying cells, and Western blot with anti‐GFP antibody demonstrated GFP‐LC3‐II conversion in GFP‐LC3‐transfected macrophages (Figure 3G).

LAP requires the NADPH oxidase 2, Nox2 for the production of reactive oxygen species (ROS).^[^ ^29^ ^]^ We found that heat‐treated tumor cells induced macrophage ROS production (Figure 3H). We applied the Nox2 inhibitor diphenyleneiodonium chloride (DPI) 1 h before macrophages were treated with the heat‐treated tumor cells. DPI reversed the production of ROS in macrophages engulfing heat‐treated cells (Figure 3H). Furthermore, we applied a Rubicon (RUBCN) siRNA to inhibit the process of LAP, and the knockdown efficiency was shown (Figure S3G, H, Supporting Information). Silencing of RUBCN was able to reduce the LC3‐II to LC3‐I ratio, indicating that macrophages engulfed dying cells through LAP (Figure 3I). We were not able to detect any effect on phagocytosis efficiency in BMDMs transfected with siRUBCN (Figure 3J), indicating that RUBCN did not exert its effects through effecting the efficiency of phagocytosis.

### 2.5 Macrophages Undergoing LC3‐Associated Phagocytosis Exhibit an M2 Phenotype and Inhibit T Cell Function

**Figure 4:**
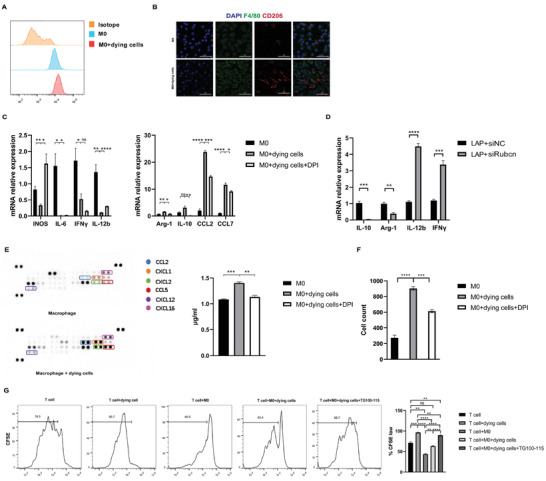
Macrophages undergoing LAP present M2 phenotype. A) CD206 expression on the surface of macrophages engulfing dying cells, measured by flow cytometry. B) Fluorescence microscopy images illustrating F4/80 (green) and CD206 (red) in the macrophages engulfing dying cells, compared with untreated macrophages. Scale Bar  =  50 µm. C) The mRNA expression of M1 marker INOS, IL‐6, IFN*γ*, IL‐12b, M2 markers Arg‐1, IL‐10, and chemokines CCL2, CCL7 (n = 3, ns > 0.05, *p < 0.05, **p < 0.01, ****p < 0.0001 by unpaired *t*‐test). D) The mRNA expression of IL‐10, Arg‐1, IL‐12b, and IFN*γ* (n = 3, ***p < 0.001, ****p < 0.0001 by unpaired *t*‐test). E) A cytokine expression array showing cytokines of cell‐free supernatants from untreated macrophages and macrophages engulfing dying cells; CCL7 ELISA of cell culture conditioned media from 3 groups. F) The chemotaxis ability of monocytes isolated from bone marrow was assessed using chemotaxis assay (n = 3, **p < 0.01, ***p < 0.001 by unpaired *t*‐test). G) Effect of TG100‐115 on the splenic CD3^+^ T lymphocyte suppression mediated by BMDMs. T cell proliferation was quantified with carboxyfluorescein succinimidyl ester (CFSE) (n = 3, ns > 0.05, *p < 0.05, **p < 0.01, ***p < 0.001, ****p < 0.0001 by unpaired *t*‐test).

We investigated the function of macrophages engulfing dying tumor cells. Compared to untreated BMDMs, flow cytometry analysis and IF of cell surface phenotypic markers CD206 confirmed the regulation of M2 markers (**Figure** **4**A,B), while qPCR for M1 (INOS, IFN*γ*, IL‐12b) and M2 (Arg‐1, IL‐10) phenotypic markers showed the up‐regulation of M2 phenotype in BMDMs engulfing dying cells (Figure 4C, Figure S4A, Supporting Information). Inhibiting LAP with siRUBCN decreased the levels of IL‐10 and Arg‐1 and increased the levels of IFN*γ* and IL‐12b (Figure 4D). We further investigated the expression of chemokines, CCL2, CCL7, CXCL1, CXCL2, CXCL16, and CCL24, which was increased after macrophages engulfed dying cells (Figure S4B, Supporting Information). Cytokine array profiles of the culture conditions showed a significant upregulation of CCL2, CXCL1, CXCL2, and CXCL16 (Figure 4E). ELISAs showed a significant upregulation of CCL7 (Figure 4E). CCL2 and CCL7 were shown to be the main chemokines recruiting monocytes, so we performed a co‐culture system with BMDMs and bone marrow cells. The bone marrow cells were seeded in the upper compartment of a Transwell insert, and the BMDMs were seeded in the lower compartment. The macrophages treated with dying cells recruited more bone marrow‐derived Ly6C^+^ monocytes in 1 h (Figure 4F). We also detected the increase of chemotaxis ability of BMDMs after engulfing dying tumor cells in the recruitment of a macrophage cell line, Raw264.7 cells, in 48 h (Figure S4C, Supporting Information).

With DPI treatment, macrophages showed higher expression of M1 genes (INOS, IL‐6, TNF*α*, IL‐12b), and decreased expression of M2 genes (IL‐10, Arg‐1) and chemokines (CCL2, CCL7) (Figure 4C). ELISAs showed lower expression of macrophages secreting CCL7 (Figure 4E). With a co‐culture system as described, DPI reduced the cell number recruited by BMDMs undergoing LAP (Figure 4F, Figure S4C, Supporting Information).

We subsequently tested the suppressive function of myeloid cells undergoing LAP on the proliferation of naïve CD3 T cells. In a co‐culture system with BMDMs and T cells, we showed that dying tumor cells stimulated the proliferation of T cell compared with untreated BMDMs, while the stimulation was abolished in BMDMs undergoing LAP, and TG100‐115 reversed the inhibitory effect of LAP (Figure 4G).

### 2.6 LC3‐Associated Phagocytosis Enhances IL‐4 Mediated Macrophage Polarization

It has been reported that inhibiting LAP reverses macrophages from M2 to M1,^[^ ^21^ ^]^ hence we further investigated the mechanism of macrophage polarization induced by LAP. The role of LAP in the polarization of macrophages is conflicting, where the modulation seems to be different according to the type of stimulant. For example, a report showed that macrophages produced higher levels of TNF, IL6, and IL‐1*β* after stimulation with *H. capsulatum*,^[^ ^18^ ^]^ whereas a different report showed that macrophages treated with dying tumor cells exhibited M2 phenotype.^[^ ^21^ ^]^

**Figure 5:**
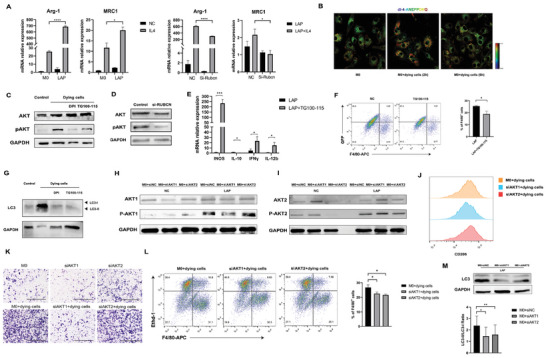
LAP in macrophages enhance IL‐4 sensitivity. A) The mRNA expression of M2 marker Arg‐1 and MRC1 (n = 3, *p < 0.05, ****p < 0.0001 by unpaired *t*‐test). B) Representative confocal images of di‐4‐ANEPPDHQ staining, red colors indicated high membrane order, whereas blue colors indicated low membrane order. C,D) Lysates from BMDMs exposed to dying cells for 1 h were analyzed by Western blot to determine the protein expression levels of phosphorylated AKT Ser473 and total AKT. E) The mRNA expression of INOS, IL‐10, IFN*γ*, and IL‐12b between 2 groups (n = 3, *p < 0.05, ***p < 0.001 by unpaired *t*‐test). F) Representative flow cytometry plots illustrating macrophages treated with TG100‐115 phagocytosis of heat‐treated GFP‐transfected Hepa1‐6 cells (n = 3, *p < 0.05 by unpaired *t*‐test). G) Lysates from BMDMs exposed to dying cells were immunoblotted with antibodies to LC3. H,I) Western blot analysis of AKT1 and AKT2 phosphorylation in BMDMs transfected with siAKT1 and siAKT2 exposed to dying cells. J) CD206 expression on the surface of BMDMs transfected with siAKT1 and siAKT2 engulfing dying cells, measured by flow cytometry. K) Migration of Raw264.7 cells (top layer of the Transwell) in a Transwell culture system in co‐culture with BMDMs transfected with siAKT1 and siAKT2 exposed to dying cells (bottom layer of the Transwell). Scale Bar  =  500 µm. L) Representative flow cytometry plots illustrating phagocytosis assay of macrophages transfected with siAKT1 and siAKT2 (n = 3, *p < 0.05 by unpaired *t*‐test). M) Lysates from BMDMs transfected with siAKT1 and siAKT2 exposed to dying cells were immunoblotted with antibodies to LC3 (n = 3, *p < 0.05, **p < 0.01 by one‐way ANOVA).

In the TME, macrophages have been shown to exhibit an M2 phenotype, driven by IL‐4 or IL‐13. To detect the effects of LAP on the polarization of macrophages, we treated BMDMs with IL‐4 in the presence or absence of dying cells. Macrophages undergoing LAP showed increased expression of IL‐4‐induced genes, Arg1 and Mrc1, and silencing of RUBCN decreased IL‐4‐induced gene expression (**Figure** **5**A). These results showed that LAP enhanced IL‐4‐mediated macrophage polarization.

A previous study has shown that membrane cholesterol efflux drives macrophage IL4‐mediated M2 reprogramming through the PI3K/AKT pathway,^[^ ^30^ ^]^ and we queried whether engulfment of heat‐treated tumor cells induced membrane cholesterol efflux. Hence, we applied a phase‐sensitive fluorescent probe, di‐4‐ANEPPDHQ, to evaluate membrane cholesterol content. This probe has a 60­nm spectral blue shift between the disordered and ordered bilayer phases, and cholesterol is more concentrated in the ordered phase domains. Di‐4‐ANEPPDHQ is a direct reflection of cholesterol content, independent of membrane‐associated peptides.^[^ ^31^ ^]^ To detect the cholesterol efflux of macrophages engulfing dying cells, we detected the staining level of di‐4‐ANEPPDHQ 2 and 6 h after macrophages were treated with dying cells, we have detected the cholesterol accumulation 2 h after LAP, and the cholesterol level was reversed 6 h after LAP, indicating cholesterol efflux during LAP (Figure 5B). Our results indicated that the engulfment of heat‐treated tumor cells induced cholesterol efflux in macrophages (Figure 5B).

### 2.7 Macrophages Undergoing LAP Activate the PI3K*γ*/AKT Pathway

It has been reported that the PI3K*γ*/AKT signaling pathway in macrophages controlled the switch between immune stimulation and suppression in inflammation and cancer.^[^ ^32^ ^]^ A recent study reported that PI3K/AKT signaling was required for IL‐4‐induced macrophage programming.^[^ ^30^ ^]^ We detected higher AKT phosphorylation in animal models and patients (Figure S5A, B, Supporting Information). Western blot analysis illustrated that after engulfment of dying cells, the levels of phosphorylated AKT increased, and silencing of RUBCN decreased the levels of phosphorylated AKT (Figure 5C,D). Inhibition of PI3K phosphorylation with PI3K*γ* inhibitors (TG100‐115) was able to reduce AKT phosphorylation (Figure 5C). For PCR, TG100‐115 increased the expression of IFN*γ*, IL‐12b, and INOS, and decreased the expression of IL‐10 (Figure 5E). The mechanisms of PI3K phosphorylation involved in LAP are unclear. It has been reported that phagocytosis of macrophages is controlled by PI3K*γ*,^[^ ^33^ ^]^ as PI‐4,5‐P2 trigger actin polymerization. To investigate the role of PI3K*γ* in LAP, we pretreated BMDMs with TG100‐115, and flow cytometry demonstrated the TG100‐115‐treated BMDMs engulfed a lower percentage of dying cells compared with DMSO‐treated BMDMs (Figure 5F). Inhibition of PI3K*γ* also reduced the LC3‐II to LC3‐I ratio (Figure 5G). Our results indicated that PI3K activity was critical for LAP‐enhanced programming.

### 2.8 Both AKT1 and AKT2 Played Roles during LAP

PI3K activation leads to AKT phosphorylation. Three AKT isoforms, AKT1, AKT2, and AKT3, have been reported. Although they share common upstream activators, increasing evidence has demonstrated differences between AKT1 and AKT2. For example, Akt1 ablation results in an M1‐like phenotype and the opposite occurs upon Akt2 ablation.^[^ ^34^ ^]^ In our study, increases in p‐Akt1 and p‐Akt2 expression were observed by Western blot analysis following incubation with dying cells, indicating that AKT1 and AKT2 both played roles during LAP (Figure 5H). To investigate the functions of AKT1 and AKT2, AKT1 and AKT2 siRNAs were applied to suppress the expression of endogenous AKT1 and AKT2, respectively (Figure S4D, Supporting Information). Flow cytometric analysis demonstrated that silencing of AKT1 partially reversed M2‐polarization (Figure 5J). With a co‐culture system as described, AKT1 suppression, but not AKT2 suppression, reduced the macrophages recruited by BMDMs undergoing LAP (Figure 5K). These results indicated that AKT1 played essential roles in LAP‐induced immunosuppression through alteration of macrophage phenotypes. On the other hand, for phagocytosis experiment, both silencing AKT1 and AKT2 reduced phagocytosis of dying cells (Figure 5L), and silencing of both AKT1 and AKT2 reduced the expression of LC3‐II, which indicated that both AKT1 and AKT2 played roles in engulfment of dying tumor cells by macrophages. Summing up our findings, the PI3K*γ*/AKT pathway exerted its effects in two ways: on one hand, the phagocytosis of macrophages was controlled by the PI3K*γ*/AKT pathway, on the other hand, after engulfing the dying cells, cholesterol efflux induced by LAP drove IL‐4‐mediated M2 reprogramming through the PI3K*γ*/AKT1 pathway in macrophages.

### 2.9 PI3K*γ* Inhibitor Enhances Anti‐Tumor Immunity after IRFA

We have shown that after IRFA, macrophages demonstrated increased infiltration into the TZ, activated the PI3K*γ*/AKT pathway, and mediated immunosuppression. We assessed whether targeting macrophages with PI3K*γ* inhibitors (TG100‐115) could suppress the progression of the residual tumors. As reported, residual tumors were resistant to anti‐PD‐1 therapy, and macrophages had an essential role in anti‐PD‐1 resistance.^[^ ^14^ ^]^ We established subcutaneous residual tumors, distant tumors, and liver orthotopic tumors in C57BL/6J mice, and performed IRFA, where H&E staining was used to evaluate the establishment of the IRFA model in orthotopic tumors (Figure S6, Supporting Information). We investigated whether TG100‐115 was able to overcome anti‐PD‐1 resistance in residual tumors (Figure S7A, Supporting Information).

**Figure 6:**
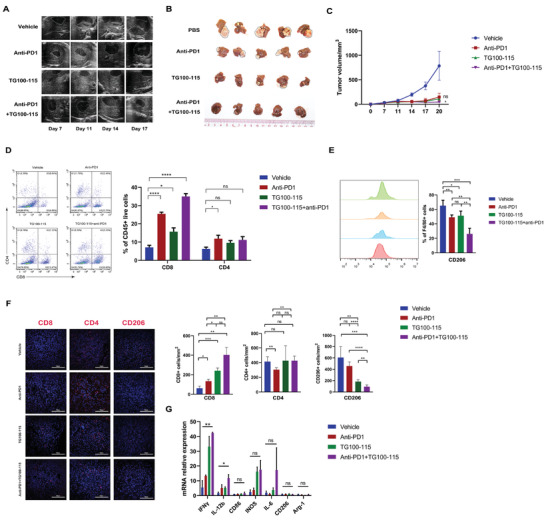
The combination of TG100‐115 and anti‐PD‐1 inhibits tumor progression in residual orthotopic tumors. A) Representative ultrasound images of tumors on day 7, 11, 14, and 17. B) Representative image of the residual orthotopic tumors. C) Growth curve of the residual tumors. D) Representative flow cytometric analysis and quantification of CD8^+^ and CD4^+^ T cell populations. E) Representative flow cytometric analysis and quantification of CD206^+^ cell populations. F) Representative IF and quantification of CD4, CD8, and CD206 in mice tumor sections in different groups. (B–F) n = 5, ns > 0.05, *p < 0.05, **p < 0.01, ***p < 0.001, ****p < 0.0001 by one‐way ANOVA. Scale Bar  =  100 µm. G) Fold change mRNA expression in tumor tissues in the 4 groups (n = 3, ns > 0.05, *p < 0.05, **p < 0.01, by one‐way ANOVA).

PI3K*γ* inhibition slowed the growth of tumors, and when synergized with anti‐PD‐1, the growth of tumors was suppressed, notably in the residual tumors and distal tumors (Figure S7B–G, Supporting Information), as well as orthotopic tumors (**Figure** **6**A–C). TG100‐115 + anti‐PD‐1 therapy also extended mice survival compared to TG100‐115 and anti‐PD‐1 (Figure S7H, Supporting Information). Analysis of tumor infiltrates lymphocytes (TILs) in dual therapy showed that TG100‐115 reversed immune suppression by reducing the ratio of CD206^+^ M2 macrophages, and improved CD8^+^ T cell numbers in residual and distant subcutaneous tumors, as well as orthotopic tumors (Figure S8A–C, Supporting Information, Figure 6D–F). We further investigated RNA expression of M1 and M2 markers in residual tumors after TG100‐115. The expression of M1‐markers (IFN*γ* and IL‐12b) was higher in TG100‐115‐treated tumors (Figure S8D, Supporting Information, Figure 6G).

## 3 Discussion

**Figure 7:**
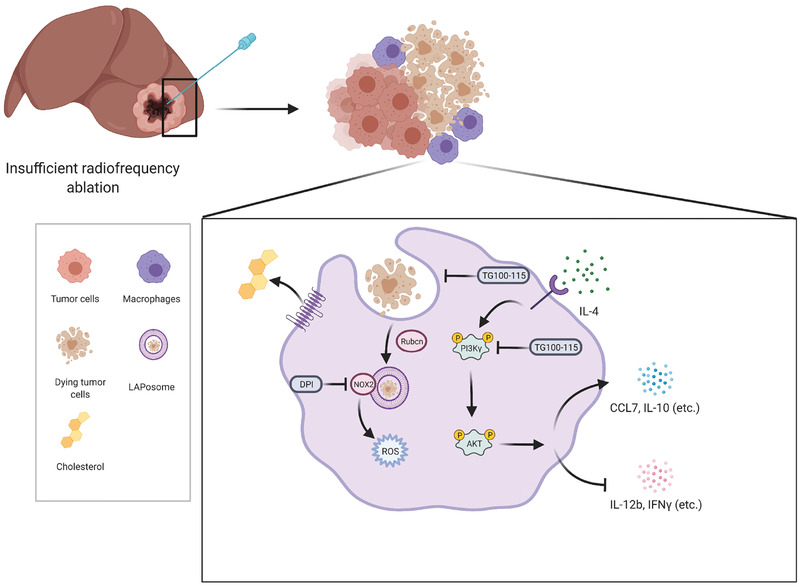
A schematic diagram of the mechanism of LAP induction in macrophages after IRFA: the macrophages in residual tumors engulf dying tumor cells, activate NOX2, and produce ROS for LAP formation. Cholesterol efflux during LAP enhances IL‐4‐mediated macrophage programming and activates the PI3K*γ*/AKT pathway. Cytokines including CCL7 and IL‐10 are produced following LAP. Simultaneously, inhibition of LAP and blockade of the PI3K*γ*/AKT pathway remodels the immunosuppressive state of macrophages. The schematic diagram is created with BioRender.com.

In our study, we found that macrophages infiltrated surrounding TZ in residual tumors in both clinical patients and animal models, and presented an M2 phenotype in animal models. Macrophages are one of the leading components in creating the immunosuppressive microenvironment. This indicated that macrophages could be a potential target in treating residual tumors after IRFA. We demonstrated that macrophages responded to dying cells in residual tumors, underwent LAP, enhanced IL‐4‐mediated programming, activated the PI3K*γ*/AKT pathway, and secreted cytokines including IL‐10, CCL2, and CCL7. In vivo and in vitro, blockade of the PI3K*γ*/AKT pathway was shown to remodel the immunosuppressive TME (**Figure** **7**).

RFA is a main curative method for liver cancer, and is characterized by low damage of tissue. However, the recurrence reported after RFA was higher than that of liver resection.^[^ ^4^ ^]^ Studies have shown if the tumor was not totally ablated, the residual tumor becomes invasive and immunosuppressive.^[^ ^13^, ^14^ ^]^ Macrophage recruitment has been observed after various forms of tissue injury. Bajpai et al. showed that after myocardial injury, CCR2^+^ but not CCR2^−^ macrophages were recruited, indicating the essential role of CCR2‐related chemokines in recruiting monocytes after tissue injury.^[^ ^35^ ^]^ In our study, we showed that macrophages that underwent LAP were the main sources of CCL2 and CCL7 and recruited monocytes into residual tumors. This process allows more macrophages to play roles in the progression of residual tumors.

LC3 expression is correlated with poor prognosis in HCC. We previously verified the existence of autophagic pathways in mice after IRFA, which promoted residual tumor proliferation after IRFA, and hydroxychloroquine (HCQ) was able to suppress the effects.^[^ ^7^ ^]^ It has been reported that LAP in TAM prevents anti‐cancer immunity, and RUBCN deficiency leads to STING‐mediated type I interferon (IFN) responses.^[^ ^18^ ^]^ It has been reported that macrophages engulf dying cells through LAP but not via canonical autophagy.^[^ ^28^ ^]^ In this study, we found that LC3B was recruited to phagosomes containing cell corpses after dying cells were engulfed by the macrophages, and only heat‐treated tumor cells induced LAP in macrophages but not in living tumor cells. Both reduction of ROS or silencing of RUBCN in macrophages reduced Arg‐1 and IL‐10 and increased IL‐12b and IFN*γ*, indicating that the immunosuppression after IRFA was related to LAP in macrophages. Recently, a study has presented a concept termed LC3‐associated endocytosis (LANDO), which is also regulated by RUBCN in Alzheimer's Disease.^[^ ^36^ ^]^ During LANDO, LC3 was recruited to endosomes containing A*β*. It has been reported that dying cells are phagocyted by macrophages,^[^ ^37^ ^]^ and our study demonstrated that macrophages engulfed heat‐treated tumor cells through LAP. This article highlights a new insight regarding the engulfment of small molecules in the TME by macrophages through LAP, which still needs further exploration.

Our study also showed that the PI3K*γ*/AKT pathway activated macrophages in response to LAP. PI3K*γ* is a key molecule mainly expressed in myeloid cells which influences immunosuppression, while blockade or silencing of PI3K*γ* activated the immune‐stimulatory effect.^[^ ^29^ ^]^ PI3K*γ* regulates innate immunity and is able to reprogram macrophages. The therapeutic effects of PI3K*γ* inhibitor are being evaluated in phase 1 clinical trial (NCT02637531).^[^ ^29^ ^]^ In our study, we have found the PI3K*γ*/AKT pathway exerts its effects in two ways: on one hand, the phagocytosis of macrophages is controlled by PI3K*γ*/AKT pathway, on the other hand, after engulfment of dying cells, cholesterol efflux induced by LAP drives IL‐4‐mediated M2 programming of macrophages through the PI3K*γ*/AKT1 pathway. Blockade of PI3K*γ* reduces the level of immunosuppressive cytokines including Arg‐1 and IL‐10, increases T cell proliferation in vitro, and overcomes resistance to anti‐PD‐1 therapy in vivo.

Our study applied three animal models: residual and distant subcutaneous tumors, and residual orthotopic tumors. PI3K*γ* inhibitors showed effectiveness in three of the models, while residual tumors showed resistance to anti‐PD‐1 therapy. As reported, the tumor‐infiltrating macrophages led to anti‐PD‐1 resistance,^[^ ^14^ ^]^ which is in line with our results, indicating the treatment prospects of combination therapy which include PI3K*γ* inhibitors and anti‐PD‐1 in clinical patients after IRFA.

Previous studies have demonstrated that interference with LAP may be a potential therapeutic strategy for tumor treatment.^[^ ^19^ ^]^ However, the methods targeting siRNAs or proteins still face challenges including safety, efficiency, and cost. In this situation, targeting the PI3K*γ*/AKT pathway may be an accessible way to treat patients after IRFA in combination with anti‐PD‐1 therapy.

In conclusion, we have uncovered a new therapeutic strategy for targeting tumor‐infiltrating macrophages in residual tumors via the PI3K*γ*/AKT pathway. PI3K*γ* inhibitor, TG100‐115, remodels the tumor immune microenvironment and promotes cytotoxic T cell‐mediated tumor immunotherapeutic effect through targeting macrophages in residual tumors. Our results introduce opportunities for new combination strategies using a PI3K*γ* inhibitor to overcome resistance to anti‐PD‐1 therapy in treating residual tumors after IRFA.

## 4 Experimental Section

### Mice and Tumor Transplantation

Wild‐type C57BL/6J mice, aged 3–4 weeks, were purchased from the Guangdong Medical Lab Animal Center. Cells from the mouse hepatoma cell line Hepa1‐6 were seeded into 75 cm^2^ cell‐culture flasks in 15 mL of Dulbecco's Modified Eagle's Medium (DMEM) with 10% fetal bovine serum (FBS) and maintained at 37 °C in a humidified atmosphere with 5% carbon dioxide. The medium was changed every two days. When the Hepa1‐6 cells were grown to ≈90% confluence, they were washed three times with PBS to remove debris and dead cells, and then trypsinized to obtain single cancer cells. The cells were then re‐suspended in a mixture of PBS and Matrigel (1:1) on ice. The final cell count was 5 × 10^7^ cells mL^−1^, and 0.1 mL of cell suspension was subcutaneously injected into the flanks of the mice.

### Establishment of Insufficient RFA Animal Model

After the tumor xenograft models were established as above, tumor growth was observed every two days. The sizes of tumors were determined by the calculation of the longest and shortest length via Vernier calipers. The tumor size was determined with the following formula:

(1) $$\begin{array}{c} V=W^{2}\times L/2 \end{array}$$

*W* and *L* refer to the width and length of tumors, respectively.

### Establishment of Pre‐IRFA Model and Post‐IRFA Model

When the length of tumor xenograft reached 10 mm, the mice were anesthetized with isoflurane (RWD Life Science Co., China), and the tumor on the left flank was removed and labeled as “pre‐RFA” group. The other tumor (on the right flank) was treated with RFA and the mouse was returned to its cage. After seven days, the RFA‐treated tumor was removed and labeled as “post‐RFA” group. RFA was performed using a bipolar RFA device (Radionics Inc., MA, USA), which was a micro radiofrequency needle with an active tip length of 10 mm. To simulate clinical IRFA, the radiofrequency needle was pierced into a non‐central location of the tumor at 3 Watts for 30 s (90 Joules).

### Establishment of Residual and Distant IRFA Subcutaneous Tumors

For treatment, the tumors were seeded at both flanks. One tumor was treated with IRFA and defined as the IRFA group, while another tumor was defined as the distant tumor without treatment.

### Establishment of Residual Orthotopic Tumors

When the tumor xenograft reached 10 mm in diameter, the tumors were harvested and non‐necrotic tissues were cut into 1 mm^3^ pieces and implanted into the left lobe of another tumor‐free mouse's liver. After 7 days, tumor volumes were evaluated by a small animal ultrasound system (VisualSonics Vevo 2100 system, Canada) and proceeded to IRFA treatment. Then, tumor‐bearing mice were randomized into vehicle and different treatment groups, and the tumor growth was monitored every three days.

### 10X Sample Processing and cDNA Library Preparation

The scRNA‐Seq libraries were prepared by a 10X Genomics Chromium Controller Instrument and Chromium Single Cell 3’ V2 Reagent Kits (10X Genomics, CA, USA). Cells were concentrated to 1000 cells µL^−1^, and approximately 17 000 cells were loaded into each channel to generate single‐cell Gel Bead‐In‐Emulsions (GEMs). For each sample, 10 000 single cells were translated into mRNA barcodes. After the real‐time (RT) step, the GEMs were broken, and the barcoded‐cDNA was purified using Recovery Agent provided by 10X followed by a Silane Dyna Bead clean‐up (Thermo Fisher, USA) and SPRI select beads (Beckman, USA), and was then amplified. The amplified barcoded cDNA was fragmented, A‐tailed, ligated with adaptors, and index PCR amplified. The final cDNA libraries were quantified using the Qubit High Sensitivity DNA assay (Thermo Fisher Scientific, USA), and the size distribution of the libraries was determined using a High Sensitivity DNA chip on a Bioanalyzer 2200 (Agilent Technologies, USA). All libraries were sequenced by HiSeq Xten (Illumina, CA, USA) on a 150 bp paired‐end run. The scRNA‐Seq data were analyzed by NovelBio Bio‐Pharm Technology using the NovelBrain Cloud Analysis Platform.

### Study Approval

Human clinical samples were obtained under the protocols using human materials that were approved by the ethics committee of the Sun Yat‐sen Memorial Hospital (Approval number: SYSEC‐KY‐KS‐2020‐212). Animal experiments were performed under guidelines approved by the Ethics Committee for the Care and Use of Laboratory Animals of the Sun Yat‐sen University (Approval number: SYSU‐IACUC‐2019‐000283).

### Statistical Analysis

Statistical analysis for zonation figures was performed using GraphPad Prism v8.0 (GraphPad Software Inc., La Jolla, CA, USA). The utilized statistical test is listed in each figure caption. Student's *t*‐test and one‐way analysis of variance (ANOVA) were applied for the comparison of quantitative data from different groups, the statistical tests were two‐sided, and *p* < 0.05 was considered as statistical significance. Data were reported as the mean ± standard error of mean (SEM).

Additional detailed materials and methods including bone marrow‐derived macrophage preparation, heat‐treated Hepa1‐6 cell preparation, phagocytosis assay, T cell suppression assay, lipid raft staining, RT reverse transcription‐polymerase chain reaction analyses, cytokine array expression analysis, generation of cell lines, transient transfection, Western immunoblotting, immunohistochemistry, IF, and flow cytometry are supplemented in Materials and Methods, Supporting Information.

## Conflict of Interest

The authors declare no conflict of interest.

## Author Contributions

L.X.D., Z.W.Y., X.Y.N., X.X.L., R.J.L., W.Y., Z.Y.S., and J.Q.C. performed research and analyzed data. L.B.M. and L.X.D. designed the study, L.X.D. and S.P.E. wrote the manuscript. All authors approved the manuscript.

## Supporting information

Supporting Information

Supplemental Video 1

Supplemental Video 2

## Data Availability

The data that support the findings of this study are available from the corresponding author upon reasonable request.
