# Supplementary material for: Targeting PI3Kγ/AKT Pathway Remodels LC3‐Associated Phagocytosis Induced Immunosuppression After Radiofrequency Ablation
Source: Adv Sci (Weinh). 2022 Jan 17;9(7):2102182. doi: 10.1002/advs.202102182 (PMC8895133; doi:10.1002/advs.202102182)
Supplement: Supplementary file 1 — Supporting Information [file ADVS-9-2102182-s003.pdf]

ADVANCED SCIENCE
Open Access

Supporting Information

for *Adv. Sci.,* DOI: 10.1002/advs.202102182

Targeting PI3Kγ/AKT Pathway Remodels LC3-Associated Phagocytosis Induced Immunosuppression After Radiofrequency Ablation

Xiaodi Liu[1,2], Wenyue Zhang[1,2], Yanni Xu[1], Xiaolin Xu[1], Qiongchao Jiang[1], Jingliang Ruan[1], Ye Wu[1], Yingshi Zhou[1], Phei Er Saw[2#], Baoming Luo[1#]

Supplementary Data

Targeting PI3Kγ/AKT Pathway Remodels LC3-Associated Phagocytosis-Induced Immunosuppression After Radiofrequency Ablation

Authors: Xiaodi Liu[1,2*], Wenyue Zhang[1,2*], Yanni Xu[1*], Xiaolin Xu[1], Qiongchao Jiang[1], Jingliang Ruan[1], Ye Wu[1], Yingshi Zhou[1], Phei Er Saw[2#], Baoming Luo[1#]

[1]Department of Ultrasound, Sun Yat-sen Memorial Hospital, Sun Yat-sen University, Guangzhou 510120, China

[2]Guangdong Provincial Key Laboratory of Malignant Tumor Epigenetics and Gene Regulation, Sun Yat-sen Memorial Hospital, Sun Yat-sen University, Guangzhou 510120, China

* These authors contributed equally to this work.

Materials and Methods

Bone marrow-derived macrophage preparation

Monocytes were generated from the tibias and femurs of C57BL/6 mice. Red Cell Lysis Buffer was applied to remove erythrocytes. A concentration of 20 ng/mL of mCSF (Novus Biologicals, USA) was added to the Dulbecco's Modified Eagle's Medium (DMEM) with 10% fetal bovine serum (FBS) and penicillin/streptomycin. After 7 days, the BMDMs were collected and the purity was verified by flow cytometry (> 95% F4/80$^+$) with FACSCelesta™ (BD Biosciences, USA) cell sorter and CytoFLEX (Beckman, USA).

Heat-treated Hepa1-6 cell preparation

Hepa1-6 cells were resuspended in DMEM and seeded at 5×10^5 cells/mL overnight. The cell culture flasks were exposed to 37°C and 60°C for 10 min. The heat-treated cells were tested with a PI / Annexin V kit. Furthermore, the cells were characterized by the viability kit with a final concentration of 20 µM of EthD-1 (Thermo Fisher Scientific, USA) and Western blot of anti-Caspase-7.

Phagocytosis assay

Macrophages were incubated with heat-treated GFP-transfected Hepa1-6 cells for 4 h in a CO_2 incubator at 37°C. After incubation, the free cells were washed away by PBS. The macrophages were labeled with anti-F4/80 antibody, and the rate of phagocytosis was characterized by flow cytometry and confocal microscopy (Zeiss LSM 800 with Airyscan, Germany).

T cell suppression assay

The isolated splenic $CD3^+$ cells were labeled with 1 mM of CFSE (Invitrogen, USA) in pre-warmed PBS for 10 min at 37°C. Then, the CFSE-labeled $CD8^+$ T cells were plated in complete DMEM media supplemented with 1 μg/mL of anti-CD3 and 1 μg/mL of anti-CD28. The BMDMs were cultured, matured, and treated with dying cells, and then the BMDMs were co-cultured with CFSE-labeled $CD8^+$ T cells. After 72 h, the cells were harvested and CFSE signal was measured by flow cytometry.

Lipid raft staining

The BMDMs were washed with pre-warmed PBS, the medium was replaced with medium supplemented with 5 μM di-4-ANEPPDHQ (US EVERBRIGHT, China). After incubation for 30 min at 37°C , the BMDMs were fixed with 4 % PFA. Fluorescence was measured by confocal microscopy (Leica SP8 STED 3X, Germany) and analyzed with FIJI software (https://imagej.net/Fiji/Downloads).

Real-time reverse transcription polymerase chain reaction analyses

Total RNA was isolated with an RNA Quick Purification kit (ESscience, China) and cDNA was synthesized with HifairTM III 1st Strand cDNA Synthesis SuperMix for qPCR (Yeasen Biotechnology, China). Quantitative amplification was performed with Hieff™ qPCR SYBR® Green Master mix (Yeasen Biotechnology, China) in a LightCycler® 480 System. Primers used were obtained from Hongxun Biotechnologies, China.

Cytokine array expression analysis

BMDMs were cultured alone or co-cultured with heat-treated Hepa1-6 cells. The supernatant was collected and centrifuged at 4000 rpm to remove cell debris. Then, the supernatant was

analyzed with a Proteome Profiler Mouse Chemokine Array Kit (R&D Systems, USA).

Generation of Cell Lines

GFP expressing Hepa1-6 cell line were produced by retroviral transduction with retroviral plasmid expressing GFP-protein. GFP-LC3 transfected macrophages were transfected retroviral plasmid expressing GFP-LC3 protein. The retroviral plasmids were obtained from Genechem, China.

Transient transfection

Rubcn siRNA, AKT1 siRNA, AKT2 siRNA, and their non-specific siRNA (NC) were constructed by IGE BIOTECHNOL. Transfection of siRNAs was performed with Lipofectamine RNA iMAX reagent (Invitrogen, USA) according to the manufacturer's instructions. Briefly, BMDMs were seeded in six-well plates with 2 mL DMEM culture medium containing 10% FBS and antibiotics. At the same time, siRNAs or NC were mixed with Lipofectamine RNA iMAX and incubated at room temperature for 15 min. Then, the complexes were transfected into BMDMs for 48 h.

Western immunoblotting

SDS-PAGE and Western blot analyses were performed. Cell lysates were prepared from BMDMs using RIPA Lysis Buffer (CWBio, China) and then subjected to 10% sodium dodecyl sulfate–polyacrylamide gel electrophoresis (SDS-PAGE). The proteins were transferred onto a PVDF membrane. After blocking with 5% skim milk (Sigma-Aldrich, USA), the membranes were incubated with the primary antibodies and then with HRP-conjugated secondary antibodies (CWBio, China). Proteins were visualized with enhanced chemiluminescence (ECL) Western blotting detection reagents (Thermo Scientific, USA) under an iBright Western Blot Imaging Systems (Thermo Scientific, USA).

Immunohistochemistry and immunofluorescence

For further histopathological analysis, before and after RFA treatment, tumors were immediately collected after being harvested. All of the tumors were fixed in 10% formalin and were paraffin-embedded. Continuous sectioning was then performed.

Immunohistochemistry (IHC) analysis was conducted using standard procedures. F4/80, CCL2, CCL7, CD4, CD8, and CD206 antibody staining was performed on sequentially cut slides. The slides were then scanned at × 40 magnification with a confocal microscope (Zeiss LSM 800 with Airyscan, Germany).

Flow cytometry

Single cell suspensions (at 10^5 cell density/tube) were acquired as previously described and were then re-suspended with PBS. Cells were stained with BUV455, V450, CD45, CD3, CD4, CD25, Foxp3, CD8a, F4/80, and CD11b at 4℃ for 30 min. CD206 antibody was stained after breaking the cell membrane with a Fix & Perm Cell Permeabilization Kit (Life Technologies, USA). Foxp3 antibody was stained for 50 min after destruction of the nuclear membrane with a Transcription Factor Buffer Set (BD Pharmingen™, USA). The cells were then washed twice with PBS and re-suspended with 200 μL of PBS. The different cellular subsets were sorted using a FACSCelesta™ (BD Biosciences, USA) cell sorter and CytoFLEX (Beckman, USA). Data was analyzed using FlowJo Version 10.2 (FlowJo LLC, USA).

Supplementary Figure Legends

Supplementary Figure 1. (A) H&E staining to evaluate the establishment of the IRFA model. (B) Sectioned tumor tissues were stained with ki67 antibody, Scale Bar = 500 μm. (C) The proportion of CD4 and Treg cells in mice tumor tissue after treatment was quantified by flow cytometry.

Supplementary Figure 2. (A) Kaplan–Meier survival analysis of patients stratified by high CCR2 expression (>50%) vs. low expression in cancer-cell–rich regions of the liver cancer (log-rank $p = 0.002$). (B-E) Pearson correlation coefficients of CCR2 mRNA expression from the TCGA LIHC dataset with immune checkpoint markers (B) CTLA4 ($r = 0.6$, $p < 0.0001$), (C) PDCD1 ($r = 0.35$, $p < 0.0001$), (D) LAG3 ($r = 0.47$, $p < 0.0001$) and (E) TIMD4 ($r = 0.39$, $p < 0.0001$).

Supplementary Figure 3. (A) 60°C-treated and 37°C-treated Hepa1-6 cells were stained with FITC-labeled anti-Annexin V antibody and PI, and analyzed by flow cytometry. (B) 60°C-treated and 37°C-treated Hepa1-6 cells were stained by a membrane-impermeant DNA

marker, EthD-1. Dying cells were labeled by the red fluorescence of EthD-1. Scale Bar = 200 μm. (C) Lysates from tumor cells exposed to different temperatures were immunoblotted with antibodies to caspase-7. (D) The purity of the macrophages was defined by expression of F4/80 by flow cytometry. (E) The phagocytosis of living and dying tumor cells in macrophages was phagocytosis of heat-treated GFP transfected Hepa1-6 cells. (F) Lysates from macrophages and tumor cells exposed to different temperatures were immunoblotted with antibodies to LC3. (F) The mRNA relative expression of RUBCN in macrophages treated with siRNAs. (G) Lysates from macrophages transfected with siRUBCN were immunoblotted with antibodies to RUBCN.

Supplementary Figure 4. (A) The mRNA expression of M1 marker INOS, IL-6, IFNγ, IL-12b, M2 markers Arg-1, IL-10 and chemokines CCL2, CCL7 between 2 groups. (B) The mRNA expression of different cytokines. (C) The chemotaxis ability of monocytes isolated from bone marrow was assessed using chemotaxis assay. Scale bar = 500 μ m. (D) The mRNA relative expression of AKT1 and AKT2 in macrophages treated with siRNAs.

Supplementary Figure 5. Representative immunohistochemistry of p-AKT, in mice tumor sections (A) and patient sections (B) both in normal tumors and tumors after IRFA were shown.

Supplementary Figure 6. (A) Anatomical analysis of liver orthotopic tumor. (B) Anatomical analysis of liver orthotopic tumor undergoing RFA. H&E staining image shows the establishment of the IRFA model in orthotopic tumor.

Supplementary Figure 7. (A) The diagram of liver cancer treatment. (B) Representative image of the residual tumors. (C) Growth curve of the residual tumors (n=5). (D) Weight of the residual tumors examined 12 days after IRFA. (E) Representative image of the distant tumors. (F) Growth curve of the distal tumor (n=5). (G) Weight of the distant tumor examined 12 days after IRFA. (H) Kaplan–Meier survival curves of mice after IRFA.

Supplementary Figure 8. (A) Representative flow cytometric analysis and quantification of $CD8^+$ and $CD4^+$ cell populations. (B) Representative flow cytometric analysis and quantification of $F4/80^+$ and $CD206^+$ cell populations. (C) Representative immunofluorescence and quantification of CD4, CD8, and CD206 in mice tumor sections in

different groups. Scale Bar = 50 μm. (D) Fold change mRNA expression in tumor tissues in the 4 groups.

Table S1: Clinical and biological features of clinical patients

Variables		Non-IRFA group n=19	Post-IRFA group n=19	*p* value
Age				
	>55 years	6	10	0.301
	≤55 years	13	11	
Gender				
	Male	15	18	0.574
	Female	4	3	
Alcohol				
	+	3	3	0.894
	-	16	18	
Cirrhosis				
	+	14	15	0.873
	-	5	6	
HBV				
	+	13	13	0.748
	-	6	8	
HCV				
	+	2	0	0.127
	-	17	21	
Tumor size				
	>50 mm^3	9	7	0.366
	≤50 mm^3	10	14	
BCLC stage B-C				
	+	9	11	0.752
	-	10	10	
Tumor nodules				
	Single	9	8	0.554
	Multi	10	13	
Satellite nodules				
	+	2	4	0.451
	-	17	17	
vascular invasion				
	+	6	6	0.836
	-	13	15	
AFP serum level				
	>300 ng/mL	4	4	0.874
	≤300 ng/mL	15	17	
Clinically significant				

portal hypertension			
+	1	1	0.942
-	18	20	

Table S2. A list of antibodies and reagents applied in this study

Reagents or antibodies	Source	Identifier
Mouse anti-CD68	Abcam	Cat#ab201340
Rabbit anti-PD-L1	Abcam	Cat#ab228462
V510 mouse anti-CD45	BD Bioscience	Cat#563891
Rabbit anti-Ki67	Abcam	Cat#ab16667
PerCP/Cyanine5.5 mouse anti-CD3	BioLegend	Cat#100217
APC mouse anti-CD4	BioLegend	Cat#100411
FITC rat anti-CD25	BD Bioscience	Cat#558689
PE rat anti-Foxp3	BD Bioscience	Cat#563101
PE mouse anti-CD8a	BioLegend	Cat#100708
Rat anti-F4/80	Abcam	Cat#ab16911
BV605 mouse anti-CD206	BioLegend	Cat#141721
PE Hamster anti-CD11c	Elabscience	Cat # E-AB-F0991F
PE/Cyanine7 mouse anti-CD11b	BioLegend	Cat#101216
Zombie Violet™ Fixable Viability Kit	BioLegend	Cat#423113
Fixable Viability Dye eFluor™ 780	eBioscience	Cat#65-0865-14
Rabbit anti-Mannose Receptor (CD206)	Abcam	Cat#ab64693
RPE-Goat anti-Rabbit IgG (H+L) Highly Cross-Adsorbed Secondary Antibody, Alexa Fluor 647	Invitrogen	Cat#C-040
FITC-Goat anti-mouse	Servicebio	Cat#GB22301
HRP Goat anti-Rabbit IgG	Servicebio	Cat#GB23303
HRP Goat anti-Rat IgG	Servicebio	Cat#GB23302
Rabbit anti-LC3B	Cell Signaling	Cat#3868
Rabbit anti-CCL7	Bioss	Cat#bs-1987R
Rabbit anti-CCL2	Bioss	Cat#bs-1955R
Rabbit anti-Phospho-Akt (Ser473)	Cell Signaling	Cat#4060

Mouse anti-Akt (pan)	Cell Signaling	Cat#2920
DCFDA / H2DCFDA - Cellular ROS Assay Kit	Abcam	Cat#ab113851
TG100-115	APExBio	Cat#A2754
CFSE	Invitrogen	Cat#65085084
GFP-Lentivirus	Genechem, Shanghai	NA
GFP-LC3 lentivirus	Genechem, Shanghai	NA
Rabbit anti-Caspase-7	Cell Signaling	Cat#9492
Di-4-ANEPPDHQ	US EVERBRIGHT	Cat#D4024
Rabbit anti-Phospho-Akt1 (Ser473)	CST	Cat#9018
Anti- mouse/Human Phospho-Akt2 (Ser473)	Bioworld	Cat#BS4720
Anti- mouse/Human AKT1 (E40)	Bioworld	Cat#BS1978
Rabbit anti-Akt2 (D6G4)	Cell Signaling	Cat#3063
Rabbit anti-Rubicon	Proteintech	Cat#21444-1-AP
Rabbit anti-GFP Tag	Proteintech	Cat#50430-2-AP
Calcein AM	Invitrogen	Cat#C3099
EthD-1	Invitrogen	Cat#E1169
InVivoMab anti-mouse PD-1	BioXcell	Cat#BE0146

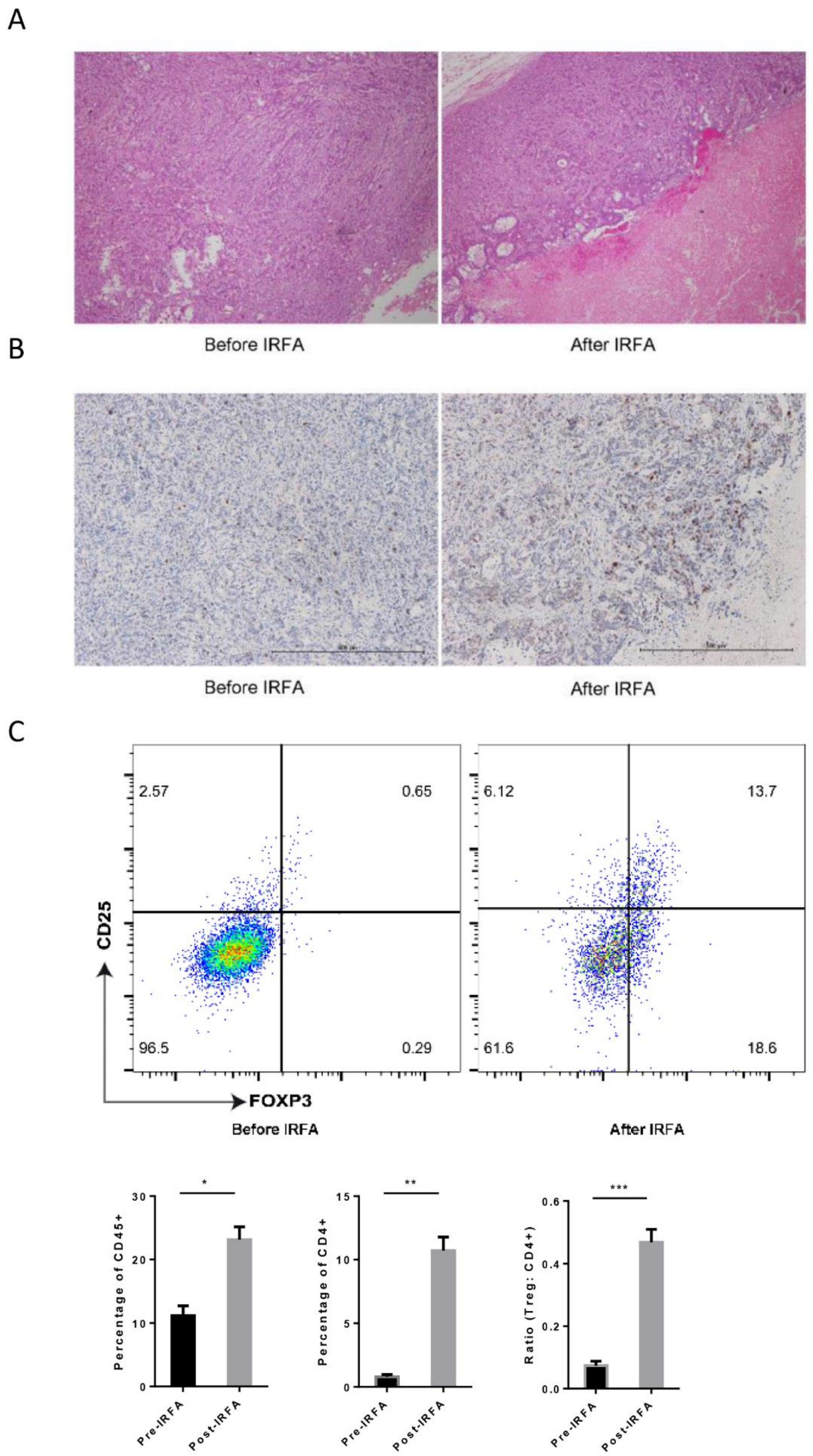

Supporting Fig. S1

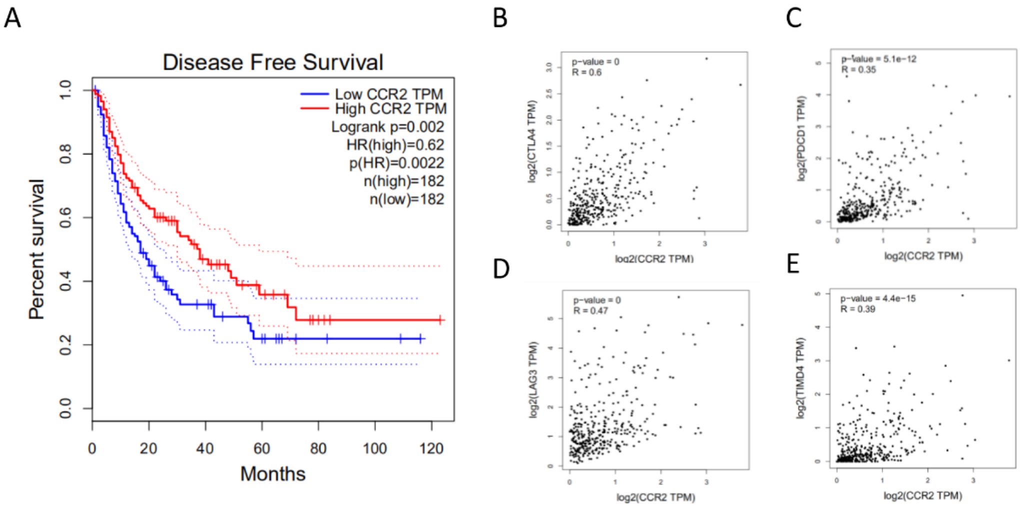

Supporting Fig. S2

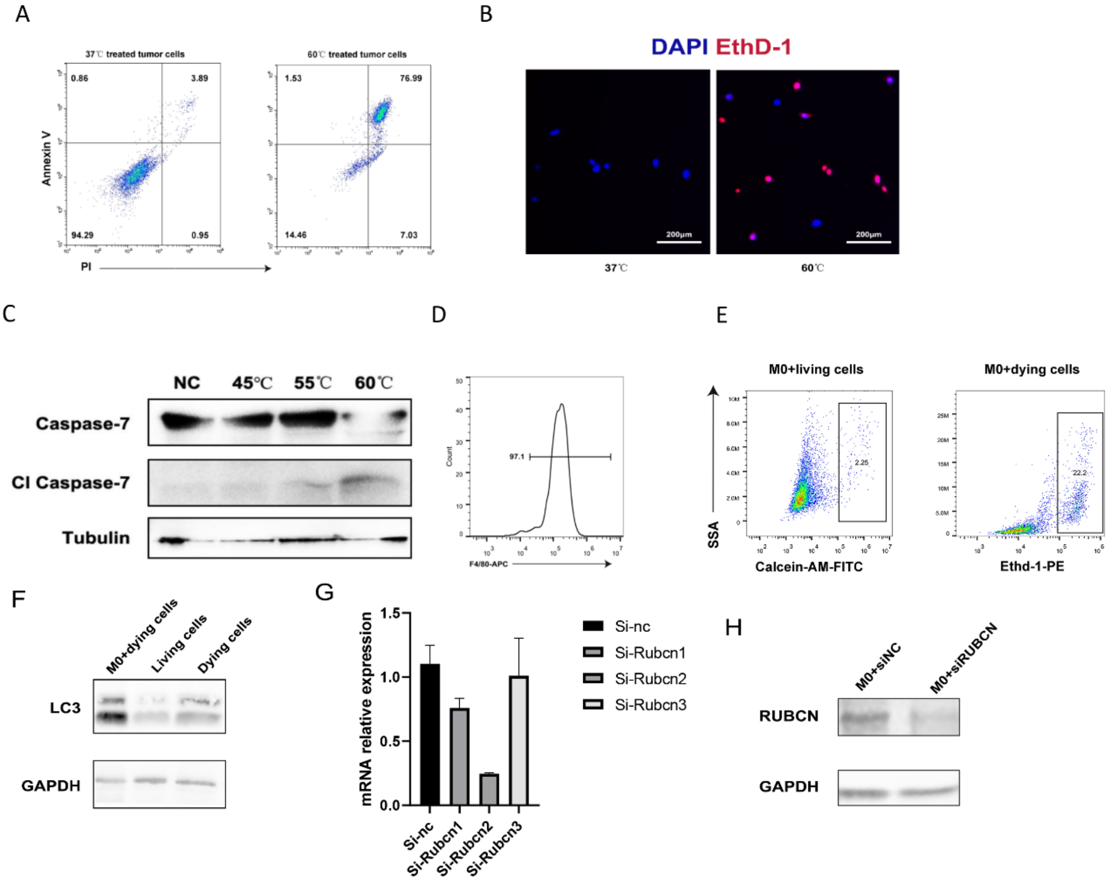

Supporting Fig. S3

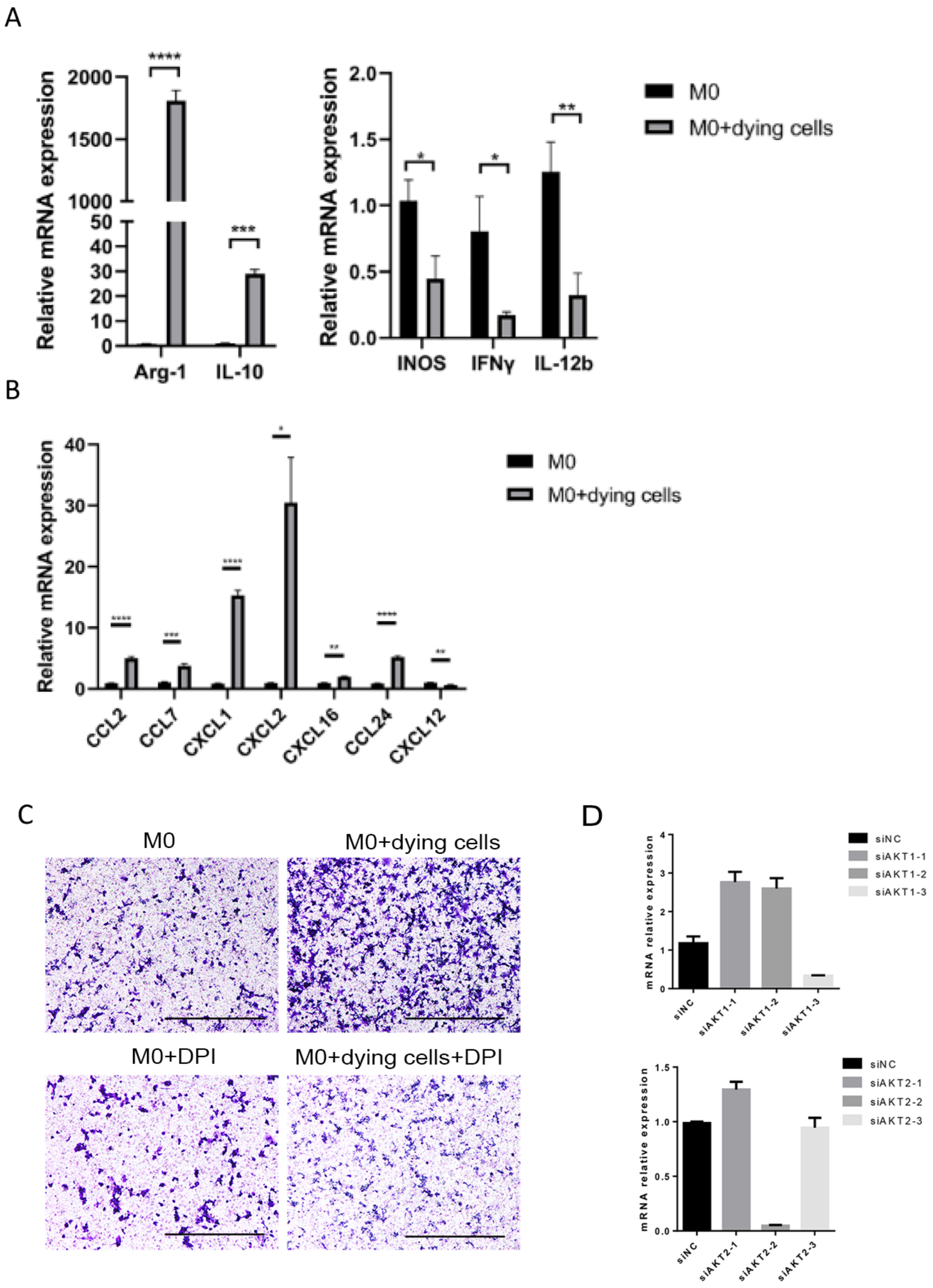

Supporting Fig. S4

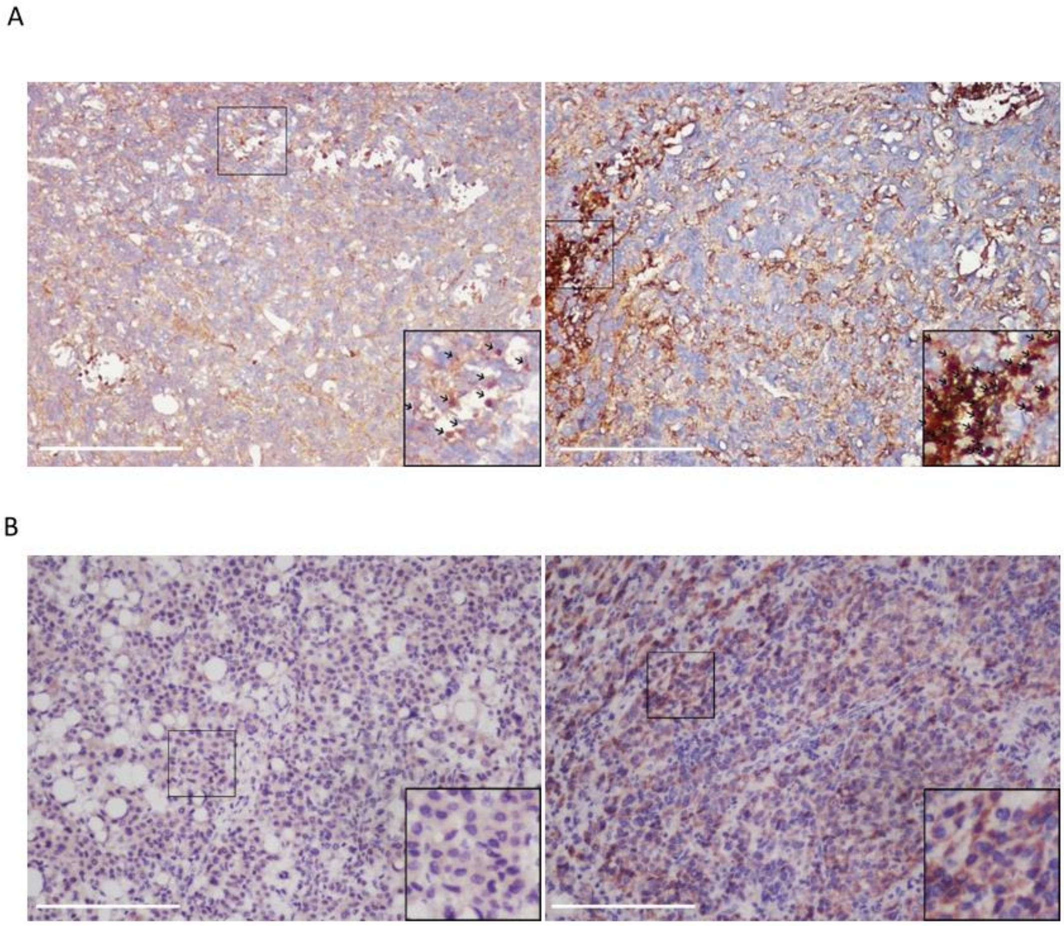

Supporting Fig. S5

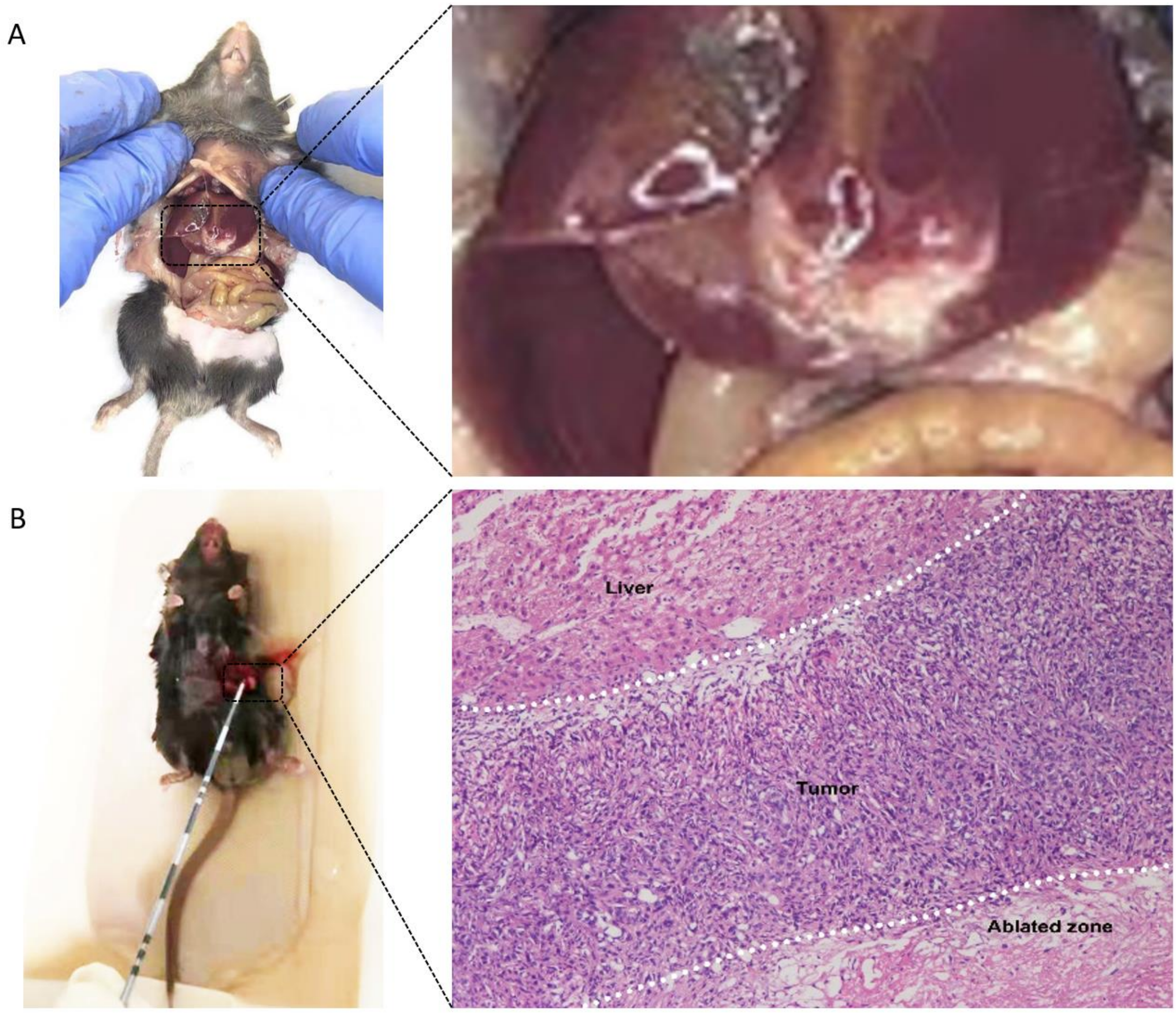

Supporting Fig. S6

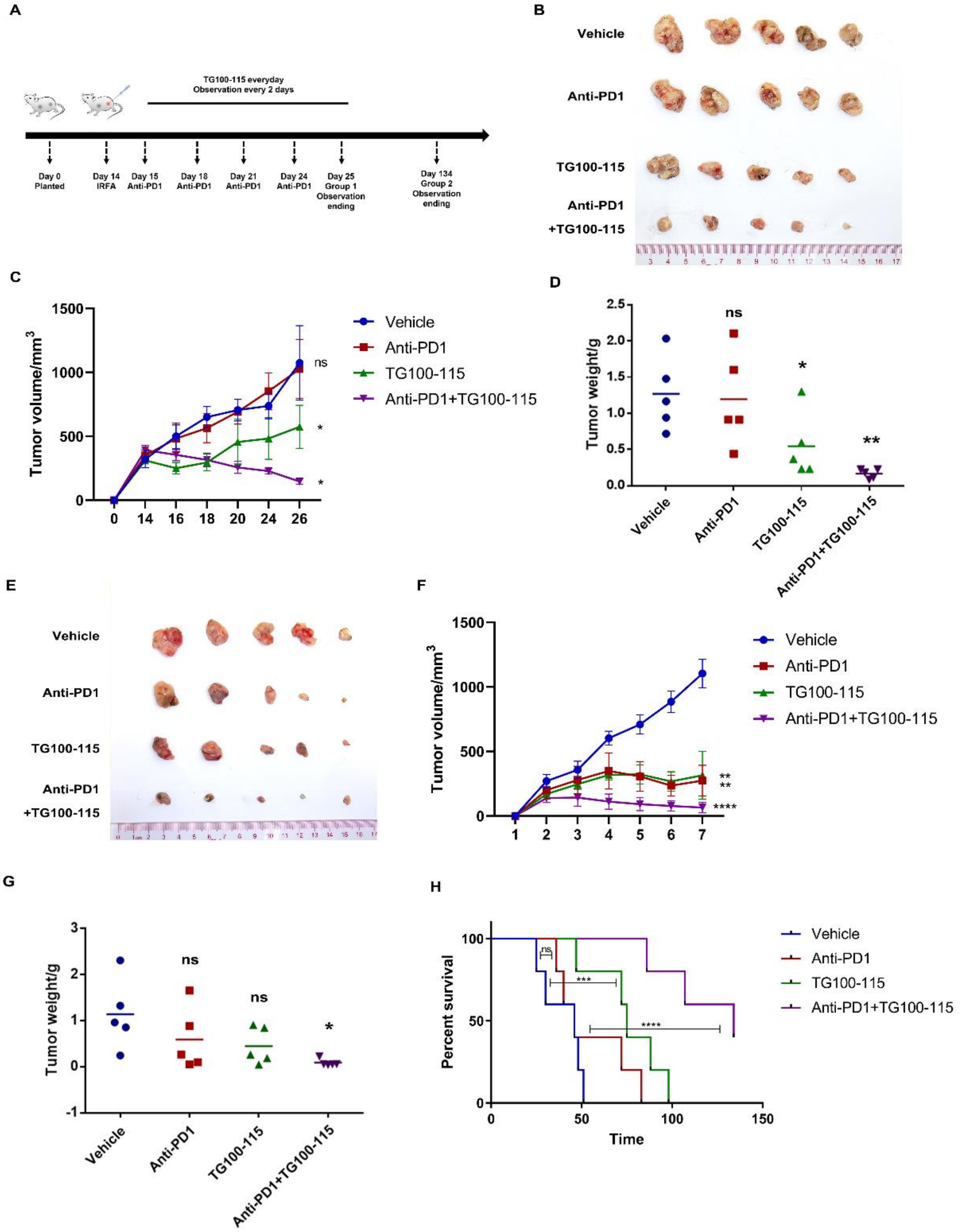

Supporting Fig. S7

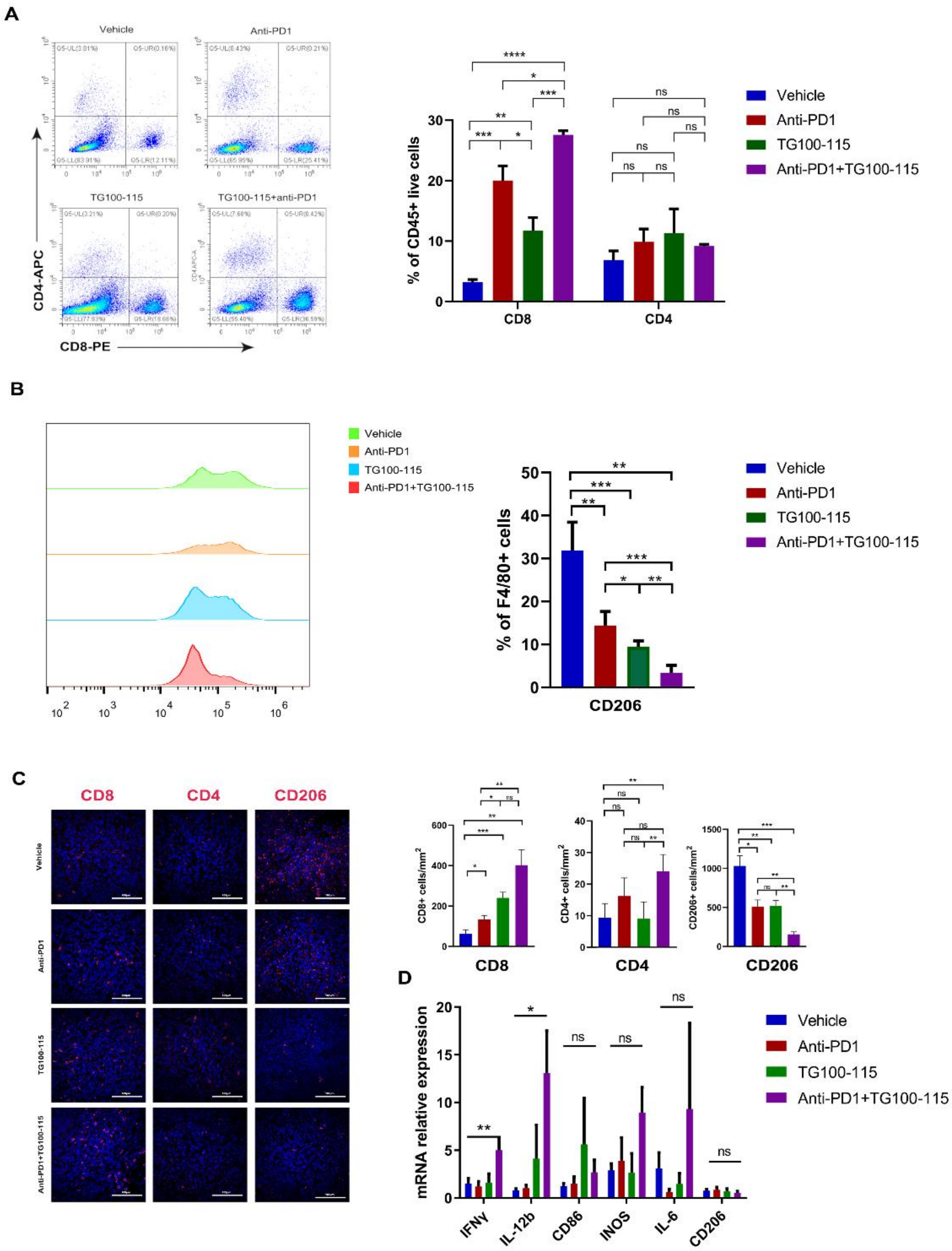

Supporting Fig. S8
